# Keeping It Simple, Transport Mechanism and pH Regulation in Na^+^/H^+^ Exchangers*

**DOI:** 10.1074/jbc.M113.542993

**Published:** 2014-03-18

**Authors:** Octavian Călinescu, Cristina Paulino, Werner Kühlbrandt, Klaus Fendler

**Affiliations:** From the Departments of ‡ Biophysical Chemistry and; § Structural Biology, Max Planck Institute of Biophysics, Max-von-Laue-Strasse 3, 60438 Frankfurt am Main, Germany

**Keywords:** Archaea, Electrophysiology, Membrane Transport, pH Regulation, Sodium Proton Exchange, EcNhaA, MjNhaP1, Cation Proton Antiporter, Solid Supported Membrane

## Abstract

Na^+^/H^+^ exchangers are essential for regulation of intracellular proton and sodium concentrations in all living organisms. We examined and experimentally verified a kinetic model for Na^+^/H^+^ exchangers, where a single binding site is alternatively occupied by Na^+^ or one or two H^+^ ions. The proposed transport mechanism inherently down-regulates Na^+^/H^+^ exchangers at extreme pH, preventing excessive cytoplasmic acidification or alkalinization. As an experimental test system we present the first electrophysiological investigation of an electroneutral Na^+^/H^+^ exchanger, NhaP1 from *Methanocaldococcus jannaschii* (MjNhaP1), a close homologue of the medically important eukaryotic NHE Na^+^/H^+^ exchangers. The kinetic model describes the experimentally observed substrate dependences of MjNhaP1, and the transport mechanism explains alkaline down-regulation of MjNhaP1. Because this model also accounts for acidic down-regulation of the electrogenic NhaA Na^+^/H^+^ exchanger from *Escherichia coli* (EcNhaA, shown in a previous publication) we conclude that it applies generally to all Na^+^/H^+^ exchangers, electrogenic as well as electroneutral, and elegantly explains their pH regulation. Furthermore, the electrophysiological analysis allows insight into the electrostatic structure of the translocation complex in electroneutral and electrogenic Na^+^/H^+^ exchangers.

## Teaser

**Background:** Na^+^/H^+^ exchangers have a pronounced pH dependence previously explained by pH sensors.

**Results:** Electrophysiological investigation of NhaP1 from *Methanocaldococcus jannaschii* (MjNhaP1), a prototype of electroneutral Na^+^/H^+^ exchangers, allowed its kinetic characterization.

**Conclusion:** The pH dependence of Na^+^/H^+^ exchangers is an inherent property of their transport mechanism.

**Significance:** The proposed mechanism of transport and pH regulation applies to all Na^+^/H^+^ exchangers.

## Introduction

Na^+^/H^+^ antiporters are crucial for survival in all organisms because they control the intracellular sodium and proton concentrations (1, 2). Most Na^+^/H^+^ exchangers belong to the superfamily of monovalent cation/proton antiporters, CPA^2^ (1). CPA1 transporters couple the transport of *n* protons to that of *n* Na^+^ ions across the membrane and are electroneutral, whereas the CPA2 subfamily includes electrogenic Na^+^/H^+^ exchangers, such as NhaA from *Escherichia coli* (EcNhaA) (1). NhaP1 from *Methanocaldococcus jannaschii* (MjNhaP1) is currently the only CPA1 exchanger for which structural information is available (3, 4). It is therefore an important prototype for the CPA1 subfamily, which includes the medically important eukaryotic NHEs.

There is general agreement that most transporters function according to the alternate access principle (5), where the substrate binding sites are alternatively exposed to the cytoplasmic and the extracellular sides of the membrane. However, the structural elements and conformational transitions vary considerably between transporter families (for reviews, see Refs. 6, 7). The mechanism of an exchanger or antiporter is in principle explained very simply (8, 9) in terms of a flexible membrane-spanning macromolecule with a common binding site for both substrates, known as a transporter. The transporter alternates between a cytoplasmically (inward) and an extracellularly (outward) open conformation if and only if the binding site is occupied. A transition between the two conformations of the empty transporter would create a “leak” that has to be avoided.

The condition that forbids a transition of the empty carrier can also be expressed in terms of thermodynamics. The energy barrier between the inward and the outward open forms of the transporter is low when the substrate is bound but high in its absence. This is a well known principle in enzyme catalysis (8, 10), where substrate binding lowers the barrier. In a conventional enzyme the barrier applies to substrate conversion. In a transporter the barrier applies to the conversion of (at least two) different conformational states. Indeed, the question of how substrate binding lowers the barrier for the conformational transition is central to an understanding of membrane transport and remains yet to be answered.

**FIGURE 1.**
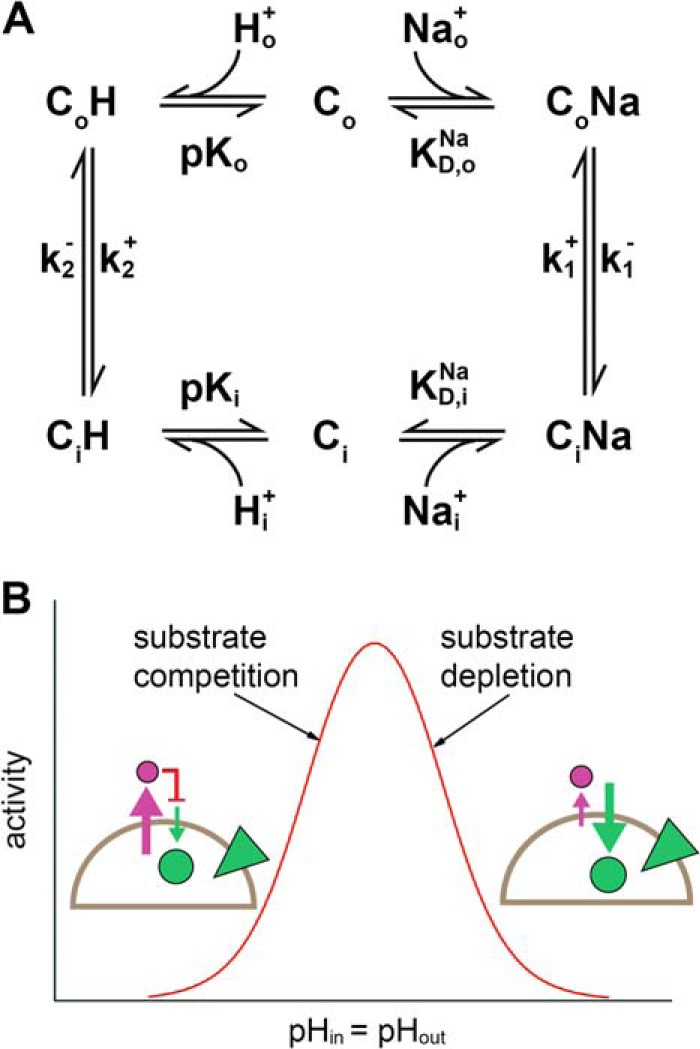
**Kinetic model for Na^+^/H^+^ exchangers.** *A*, kinetic model of Na^+^/H^+^ exchange. The substrate Na^+^ or H^+^ binds to the outward open (C*_o_*) or inward open (C*_i_*) form. Substrate binding is assumed to be in rapid equilibrium described by the parameters p*K_o_* = p*K_i_* as well as *K*_*D*,*o*_^Na^ = *K*_*D*,*i*_^Na^. The reorientation of the carrier takes place with the rate constants *k*_1_^+^ = *k*_1_^−^ and *k*_2_^+^ = *k*_2_^−^. Directions + and − were chosen arbitrarily to correspond to the physiological forward transport direction of EcNhaA. *B*, typical activity profile of a Na^+^/H^+^ exchanger calculated according to the steady-state solution of the kinetic model under symmetrical pH conditions (identical pH inside and outside) and an inward directed sodium gradient. Note that at physiological conditions the relevant mechanism of down-regulation is substrate depletion for transporters of the CPA1 family and substrate competition for transporters of the CPA2 family. *Schematic* in *B* shows the transport direction of H^+^ (*purple circle* and *arrow*) and Na^+^ (*green circle* and *arrow*), as well as the direction of the Na^+^ gradient (*green triangle*).

A striking feature of all Na^+^/H^+^ exchangers is their pronounced pH dependence. Specialized protein regions, referred to as “pH sensors” or “proton modifiers” have been suggested to account for the strong pH dependence (2). This concept has recently been challenged by the finding that EcNhaA is active at cytoplasmic pH 5 (11). It was shown that the substrate dependence of EcNhaA is explained by a simple kinetic model with a single substrate binding site, which is alternatively occupied by one Na^+^ or two H^+^ (11). On this basis it was proposed that the pH dependence of Na^+^/H^+^ exchangers is a unique property of their transport mechanism. An attractive feature of the model is that Na^+^/H^+^ exchangers (Fig. 1*A*) are inherently down-regulated at alkaline or acidic pH (Fig. 1*B*) and that there is no intrinsic requirement of any “pH sensing” regions other than the substrate binding site. This mechanism prevents excessive acidification of the cytoplasm in the case of the CPA2 exchanger EcNhaA as well as excessive alkalinization in the case of the CPA1 transporter MjNhaP1.

To investigate the pH regulation of CPA1 exchangers we have undertaken an electrophysiological analysis of MjNhaP1. CPA1 Na^+^/H^+^ exchangers have so far not been characterized by such methods because their net transport reaction is electroneutral so that steady-state currents cannot be recorded. However, we were able to capture the Na^+^ translocation step by solid-supported membrane (SSM)-based electrophysiology under conditions where the compensating H^+^ transport step does not occur. In this way, we tested and verified the previously described kinetic model for Na^+^/H^+^ antiporters for a CPA1 antiporter and obtained new insights into the substrate translocation complex and the electrogenic nature of partial reactions in the MjNhaP1 transport cycle.

## EXPERIMENTAL PROCEDURES

### 

#### 

##### Plasmids and Bacterial Strains

A MjNhaP1 construct containing C-terminal Myc and His tags in the pTrcHis2-Topo vector was used (4). Point mutations were generated using the QuikChange II Site-directed Mutagenesis kit (Agilent Technologies) and the following mutagenic primers: MjNhaP1_N160A_s (GTTAGAGGCGGAGAGTATCTTTgcCGACCCATTGGGAATAGTTTC) and MjNhaP1_N160A_as (GAAACTATTCCCAATGGGTCGgcAAAGATACTCTCCGCCTCTAAC). The MjNhaP1 D161A mutant was kindly provided by Prof. Zeilinger (12) and was obtained using the primers with MjNhaP1_D161A_s (TATTCCCAATGGGgCGTTAAAGATA) and MjNhaP1_D161A_as (TATCTTTAACGcCCCATTGGGAATA).

For protein production, *E. coli* BL21(DE3) cells were used.

##### Overexpression, Purification, and Reconstitution of MjNhaP1

MjNhaP1 WT and mutants were overexpressed in *E. coli* BL21(DE3) and purified using Ni^2+^ affinity chromatography as described previously (4). Reconstitution of purified protein was performed essentially as described previously (4) at lipid to protein ratios of 10 or 50 (w/w) and to the indicated lipid concentration.

##### Na^+^ Efflux in Proteoliposomes

MjNhaP1 proteoliposomes were prepared at lipid to protein ratios of 50 and resuspended in Na^+^ loading buffer (10 mm MES, 10 mm HEPES, 10 mm Tris, pH 8, 300 mm NaCl, 5 mm MgCl_2_) at 15 mg/ml lipid concentration. 10 μl of liposomes was diluted in efflux buffer (10 mm MES, 10 mm HEPES, 10 mm Tris, 600 mm mannitol, 5 mm MgCl_2_) titrated to various pH values with Tris or HCl, and mixed using magnetic stirring for 20 s. The suspension was added to a chromatographic column containing 0.5 ml of the strong cation exchange resin Dowex 50WX8 (*N*-methyl-d-glucamine^+^) (Dow Chemical Company) and passed through the column using a flow rate of 12 ml/min generated by a HiLoad Pump P-50 (GE Healthcare) to remove sodium that was transported out of the liposomes. Proteoliposomes were eluted by addition of water, and the eluate was diluted to 5 ml. Na^+^ concentrations were determined by atomic absorption spectroscopy using an AAnalyst 100 system (PerkinElmer Life Sciences). To verify that determined concentrations corresponded to Na^+^ concentrations inside the proteoliposomes, proteoliposomes solubilized in 0.05% Triton X-100 were subjected to the same treatment. The Na^+^ concentrations determined with solubilized proteoliposomes were indicative of the amount of Na^+^ that could not be removed by the ion exchanger and were subtracted from all measured values. A base level of Na^+^ at which the efflux of Na^+^ through MjNhaP1 is considered 0 was taken for the efflux at pH 8. The efflux was considered 100% at outside pH 5, and all measurements were normalized to this value.

##### SSM-based Electrophysiology

SSM measurements were performed as described previously (13, 14). 27.5 μl of lipid to protein ratios of 10 proteoliposomes (at 3.33 mg/ml lipid concentration) were added to the SSM sensor containing an octadecanethiol/phospholipid hybrid bilayer adsorbed on a gold surface and allowed to incubate for at least 1 h. SSM measurements were performed according to a single solution exchange protocol (nonactivating solution, 0.5 s; activating solution, 0.5 s). Currents were amplified with a current amplifier set to a gain of 10^9^ V/A and a rise time of 10 ms. All measurement solutions for Na^+^ and Li^+^ concentration jumps contained 50 mm MES, 50 mm HEPES, 50 mm Tris, 200 mm choline chloride, 5 mm MgCl_2_, and 1 mm dithiothreitol and were titrated to the desired pH with Tris or HCl. In addition, nonactivating solutions contained a further 100 mm choline chloride, whereas activating solutions contained *x* mm NaCl (or LiCl) and (100 − *x*) mm choline chloride. The measurement solutions for pH jumps all contained 20 mm MOPS, 5 mm MgCl_2_, 200 mm choline chloride, 1 mm dithiothreitol and were titrated to the desired pH with Tris. Additionally, solutions contained either a further 100 mm choline chloride or 100 mm NaCl.

##### Correction of Transient Currents

Two methods were used to correct the recorded SSM transient currents for effects due to the interaction of the substrate concentration jumps with the lipid membrane. First, transient currents recorded for the same substrate concentration jumps on empty liposomes were subtracted from the transient currents recorded on MjNhaP1 proteoliposomes. For pH jumps the solution exchange effect was recorded on the same sensor by adding 100 mm Na^+^ to the activating and nonactivating solutions to block the exchanger in the Na^+^-bound form. Alternatively, amplitudes of the transient currents for Na^+^ and Li^+^ concentration jumps could be corrected directly by subtraction of the amplitudes of the same substrate concentration jump on empty liposomes, yielding results comparable with the subtraction of the current traces.

##### Kinetic Analysis and Numerical Methods

Based on the kinetic model shown in Fig. 1*A* steady-state and pre-steady-state solutions were determined. An analytical steady-state solution was calculated as described previously (11) using Mathcad (Parametric Technology Corporation, Needham, MA). The turnover calculated by this approach was used for the simulations in Fig. 6. For the fit in Fig. 4 a numerical solution was determined by solving the defining differential equations of the kinetic model shown in Fig. 1 using Berkeley Madonna (version 8.3.18; Berkeley Madonna Inc., University of California, Berkeley, CA).

##### Setting Up the Differential Equations

Intermediates, substrate concentrations, and rate constants are labeled as *o* = out (periplasmic) or *i* = in (cytoplasmic). In the case of the conformational transitions (*k*_1_, *k*_2_) forward (+) was arbitrarily chosen as the physiological forward transport direction of EcNhaA.

The system of coupled differential equations describing the kinetic model in Fig. 1*A* is

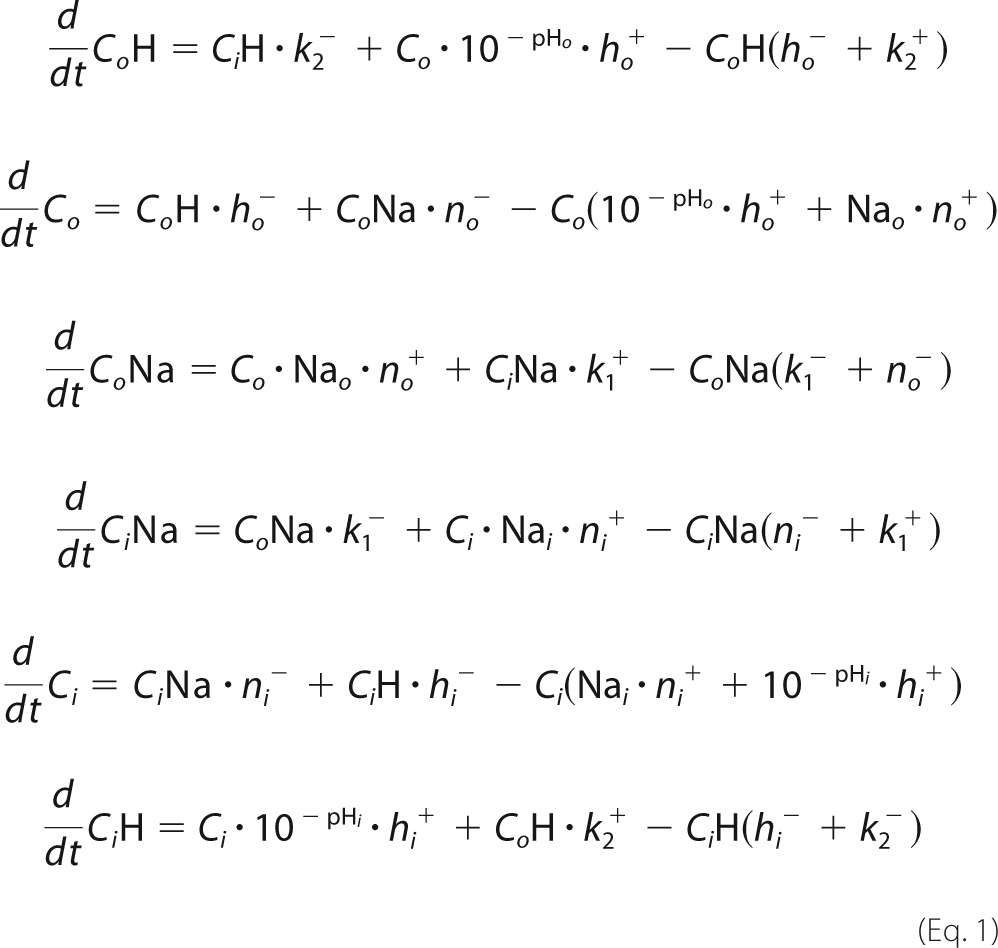
 The system of differential equations was numerically solved using the initial conditions at the given pH (for Na^+^ and pH jump experiments the initial pH was the same inside and outside and is set to pH_i_)

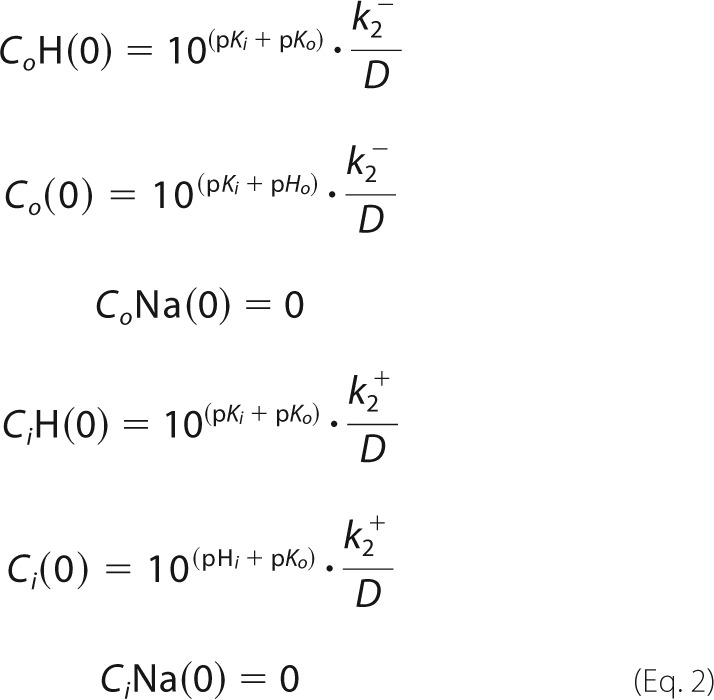
 with

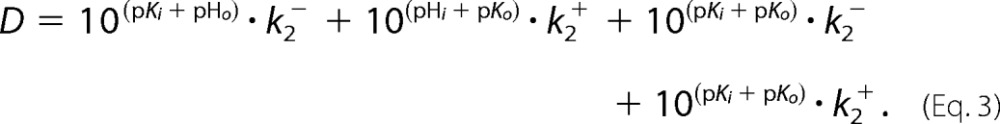


##### Calculation of the Transient Currents, Numerical Pre-steady-state Solution

From the intermediate concentrations the time-dependent transporter current *I_T_*(*t*) was calculated according to

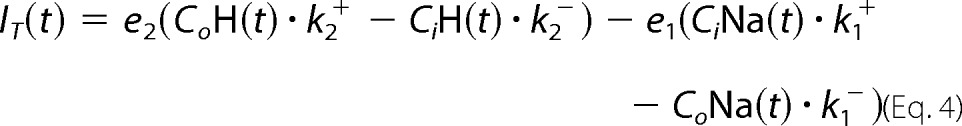
 The parameters *e*_1_ and *e*_2_ are the displaced charge in the Na^+^ and the H^+^ translocation reaction.

Solution exchange on the surface of the SSM has a time constant of ∼ 5–15 ms (13). This affects the time course of the transient current. To take into account the limited speed of solution exchange we used a third-order low pass filter with a time constant of 2.2 ms. This reproduced the transient currents measured on the SSM and corresponds to a time constant of the solution exchange of 6 ms. This yields the measured current *I*(*t*).

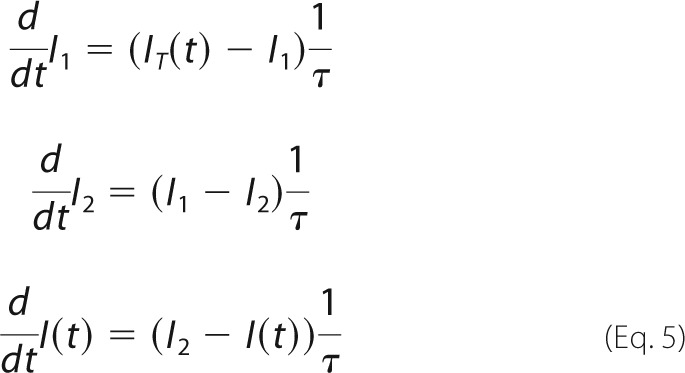
 The substrate binding and release rate constants are chosen in a way to ensure fast (<0.1 ms) relaxation of the binding equilibria. In addition they have to satisfy the relationships defining the preset p*K* and *K_D_* values (*x* = *o* or *i*).

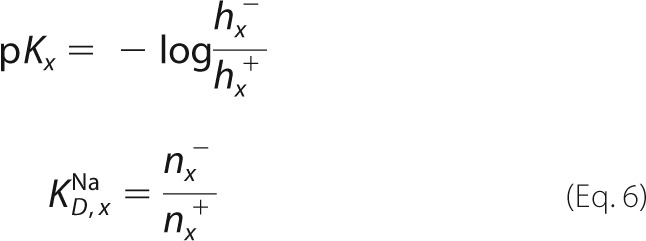
 A symmetrical kinetic model was chosen throughout this report.

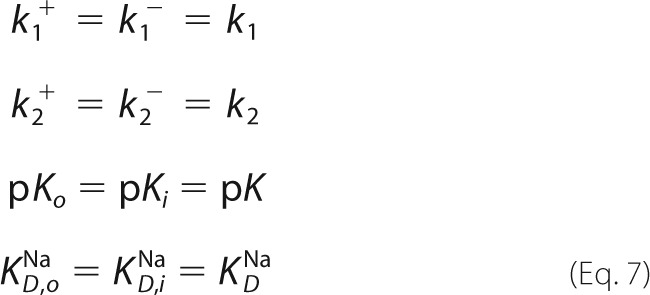
 The displaced charge for MjNhaP1 was set to

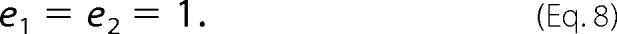


##### Calculation of Turnover, Numerical Steady-state Solution

This may be obtained using the same system of differential equations by calculating the current at sufficiently large time values. In this case the charge parameters were set to

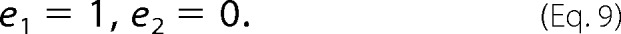
 The results of this calculation represent steady-state activity or turnover of the transporter and were used in Fig. 4*C*.

## RESULTS

### 

#### 

##### Substrate Concentration Jumps Induce Transient Currents in MjNhaP1

**FIGURE 2.**
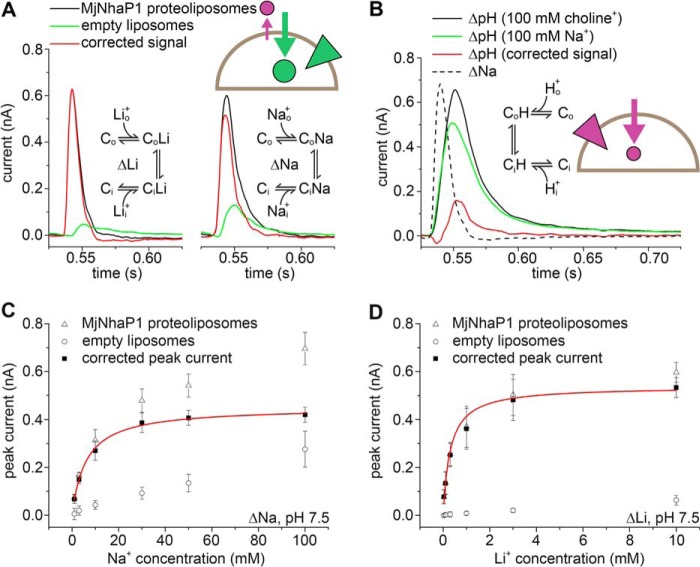
**Substrate concentration jumps performed on MjNhaP1 proteoliposomes induce a transient current.** *A*, transient currents recorded after a 0–10 mm Li^+^ concentration jump (ΔLi) or a 0–50 mm Na^+^ concentration jump (ΔNa) at pH 7.5 on MjNhaP1 proteoliposomes or empty liposomes. Subtraction of the two signals shows the corrected transient current (in *red*) generated by MjNhaP1. *B*, transient currents recorded after a jump from pH 7.5 to pH 6.5 (ΔpH) on MjNhaP1 proteoliposomes, with either choline^+^ or Na^+^ as a background. Subtraction of the two signals shows the corrected transient current generated by MjNhaP1 (in *red*). For comparison the trace of a 50 mm Na^+^ concentration jump at pH 7.5 on the same sensor (ΔNa) is also shown. The time scale in *A* and *B* is determined by the solution exchange protocol: the valve switches at *t* = 0.5 s, and the activating solution reaches the SSM ∼ 35 ms later. *Insets* indicate the specific partial reactions involved in the particular concentration jumps. *Schematics* show the transport direction of H^+^ (*purple circle* and *arrow*) and Na^+^ (*green circle* and *arrow*), as well as the direction of the Na^+^ (*green triangle*) or H^+^ (*purple triangle*) gradients. *C* and *D*, substrate dependence of transient currents at pH 7.5. Dependence of the peak currents was recorded for concentration jumps of Na^+^ (ΔNa, *C*) or Li^+^ (ΔLi, *D*) at pH 7.5. Peak currents recorded on MjNhaP1 proteoliposomes could be corrected for solution exchange effects by subtraction of peak currents recorded on empty liposomes. *Red lines* are hyperbolic fits of the corrected peak currents. Fit parameters are given in Table 1. *Error bars*, S.D.

Although the overall transport cycle of MjNhaP1 is electroneutral, the partial reactions of Na^+^ or H^+^ translocation (depicted in the *insets* in Fig. 2, *A* and *B*) are electrogenic. We were able to separate the Na^+^ and H^+^ translocation reactions by SSM-based electrophysiology and thus to analyze the transport mechanism by monitoring the two partial ion translocation steps of MjNhaP1.

Reconstituted MjNhaP1 was activated by fast Na^+^, Li^+^, or H^+^ concentration jumps. MjNhaP1 has been found to be inactive at pH above 7.5 (4, 15). Using conditions where the pH was kept at 7.5 on both sides of the membrane, proteoliposomes adsorbed to the SSM gave rise to a fast positive transient current in response to Na^+^ (ΔNa) or Li^+^ (ΔLi) concentration jumps (Fig. 2*A*). To correct for the solution exchange effect due to unspecific interactions between cations and the lipid surface of the SSM (16), control experiments were conducted with empty liposomes. The transient currents obtained with empty liposomes showed a 4–10-fold lower signal, which could be subtracted from the currents measured with proteoliposomes. The resulting protein-dependent currents had high amplitudes ranging between 0.4 and 0.75 nA (Fig. 2*A*). At saturating substrate concentrations, the amplitudes of the currents recorded for Na^+^ and Li^+^ on the same sensor were similar, indicating that the same charge was being displaced in both cases.

A transient current was also recorded upon a pH jump (ΔpH) in the absence of sodium using an SSM sensor with adsorbed MjNhaP1 proteoliposomes (Fig. 2*B*). Because the pH jump effects varied significantly between different sensors, we applied a different correction method. To block the transporter in a Na^+^-bound form, 100 mm choline^+^ in both activating and nonactivating solutions was replaced by 100 mm Na^+^. By this approach the solution exchange effect for the respective pH jump was recorded for each individual sensor. This was then subtracted from the transient currents recorded for pH jumps in the absence of Na^+^. As observed for Na^+^ and Li^+^ concentration jumps (Fig. 2*A*), a pH jump from pH 7.5 to pH 6.5 resulted in a positive transient current (Fig. 2*B*). The recorded transients indicated that in MjNhaP1, translocation of H^+^ is slower than for Na^+^ or Li^+^. For a pH jump from pH 9.5 to pH 8.5 (data not shown), identical currents were recorded in the absence or presence of Na^+^ ions, indicating that no H^+^ are translocated. This is consistent with a p*K* of 6.8 as determined in our kinetic analysis (see below), which predicts a fully deprotonated binding site in the pH range from 8.5 to 9.5.

The experiments shown in Fig. 2 characterize the partial Na^+^ and H^+^ translocation steps as electrogenic, revealing a positive charge displacement. Integration of the corrected currents allowed calculation of the displaced charge and the comparison between the charge displaced in Na^+^ concentration jumps and pH jumps performed on the same SSM sensor. In an electroneutral transporter, an equal charge is expected to be displaced in the Na^+^ and H^+^ translocation steps. However, the displaced charge observed for a pH jump was only 48 ± 14% of the charge displaced in the Na^+^ jump experiment. We ascribe this to insufficient saturation of the H^+^ binding site in the pH jump from 7.5 to 6.5. Indeed, a control calculation using the kinetic model for these experimental conditions yielded a similar result of 64%.

##### pH Dependence of MjNhaP1

**FIGURE 3.**
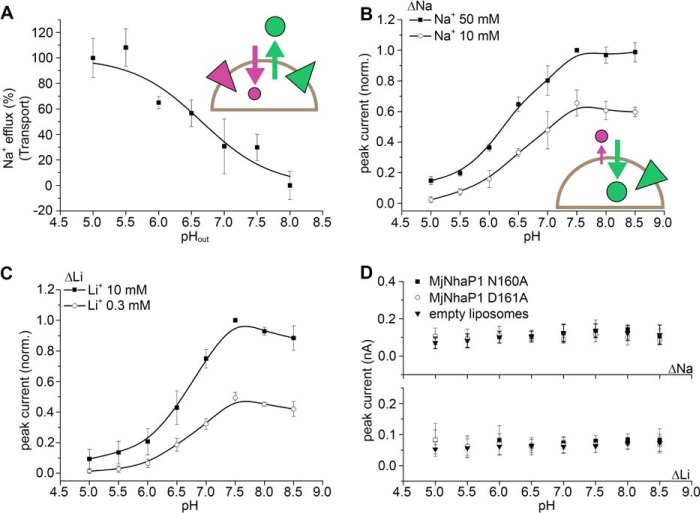
**pH response of MjNhaP1.** *A*, measurement of Na^+^ efflux with MjNhaP1 proteoliposomes by atomic absorption spectroscopy. Data points are the average of three individual efflux experiments for every pH value ± S.D. (*error bars*). *B* and *C*, pH dependence of peak currents recorded for concentration jumps of 10 and 50 mm Na^+^ (ΔNa, *B*) and 0.3 and 10 mm Li^+^ (ΔLi, *C*) on MjNhaP1 proteoliposomes. Peak currents were corrected and normalized to the peak current recorded for a concentration jump of 50 mm Na^+^ (ΔNa, *B*) and 10 mm Li^+^ (ΔLi, *C*) at pH 7.5. *D*, pH dependence of peak currents recorded after 50 mm Na^+^ or 10 mm Li^+^ concentration jumps for proteoliposomes containing the MjNhaP1 N160A and MjNhaP1 D161A mutants. Data shown in *B* to *D* are the average of recordings using three individual sensors ± S.D. *Schematics* in *A* and *B* show the transport direction of H^+^ (*purple circle* and *arrow*) and Na^+^ (*green circle* and *arrow*) as well as the direction of the Na^+^ (*green triangle*) or H^+^ (*purple triangle*) gradients. *Solid lines* are provided as guides.

**FIGURE 4.**
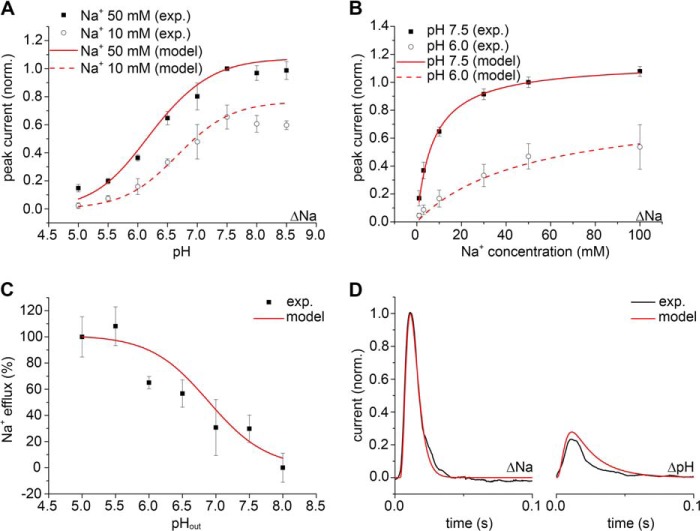
**The kinetic model of antiport describes the properties of MjNhaP1.** Simultaneous fit of the experimental data for MjNhaP1 is shown using the numerical pre-steady-state and steady-state solutions of the kinetic model in Fig. 1*A*. Adjusted fit parameters were p*K_o_* = p*K_i_* = p*K* = 6.8, *K*_*D*,*o*_^Na^ = *K*_*D*,*i*_^Na^ = *K*_*D*_^Na^ = 0.014 m, *k*_1_^+^ = *k*_1_^−^ = 350 s^−1^, *k*_2_^+^ = *k*_2_^−^ = 69 s^−1^ and the filtering time constant τ = 0.0022 s. *A*, fit of the normalized amplitude of the transient currents as a function of pH. *B*, fit of the normalized amplitude of the transient currents as a function of the Na^+^ concentration. *C*, fit of the normalized Na^+^ efflux as a function of pH. *D*, fit of the time course of the normalized transient current for a Na^+^ concentration jump (ΔNa) of 50 mm at pH 7.5 and for a pH jump (ΔpH) from pH 7.5 to 6.5 at 0 Na^+^.

The pH dependence of MjNhaP1 transport has previously been investigated in everted vesicles by acridine orange dequenching (15), an assay that only works above pH 6. To examine transport activity in the pH range 5–8 (as for the SSM experiments), we monitored Na^+^ efflux by atomic absorption spectroscopy. MjNhaP1 was reconstituted into proteoliposomes containing 300 mm Na^+^ at pH 8. Transport was initiated by adding proteoliposomes to Na^+^-free buffer at a defined pH (Figs. 3*A* and 4*C*). Note that in the acridine orange assay the pH in everted vesicles was adjusted at the Na^+^ uptake side (outside), whereas in our experiments the pH remained unchanged at the Na^+^ uptake side (pH 8 inside), preventing substrate competition for the same binding site. The measured pH profile indicated a plateau of high Na^+^/H^+^ exchange activity at pH ≤5.5. Transport decreased with increasing outside pH, with the lowest observed transport activity at pH 8.

As pointed out above, the full steady-state turnover cycle of an electroneutral transporter cannot be monitored by electrophysiology. Thus, SSM-based electrophysiological measurements with the electroneutral MjNhaP1 cannot yield a pH profile of steady-state transport activity as shown in Fig. 3*A*. However, under conditions where the H^+^ translocation step is slowed down or absent, for example at high pH, the partial electrogenic Na^+^ or Li^+^ translocation step can be monitored and analyzed by SSM-based electrophysiology (Fig. 3, *B* and *C*). The resulting transient currents recorded upon Na^+^ or Li^+^ concentration jumps on the SSM sensor (Fig. 3, *B* and *C*) show an inverse pH profile compared with Fig. 3*A*. Hence, at low pH, where transport activity is high and substrate competition takes effect, transient currents are small, whereas strong currents are recorded at high pH, due to H^+^ depletion. For the Na^+^ and Li^+^ concentration jumps shown in Fig. 3, *B* and *C*, currents reached a plateau level at pH ≥7.5.

##### MjNhaP1 D161A and N160A Mutants

CPA1 antiporters contain an Asn-Asp (ND motif) at the putative binding site, whereas the electrogenic CPA2 transporters have two Asp residues (DD motif) instead. It has been shown that mutation of the aspartates results in complete loss of activity, indicating that they are essential for transport in EcNhaA (2) and MjNhaP1 (12). We confirmed that replacement of Asp-161 in MjNhaP1 with alanine abolished the MjNhaP1-specific transient currents. Following either Na^+^ or Li^+^ concentration jumps performed using MjNhaP1 D161A proteoliposomes, the currents recorded were similar in amplitude and shape to those recorded for empty liposomes (Fig. 3*D*). Interestingly, a similar effect was found following replacement of Asn-160 with alanine (Fig. 3*D*). Thus, we were able to show that this highly conserved Asn residue found in CPA1 members (4) is equally essential for Na^+^ translocation in MjNhaP1.

Note that both investigated mutants correctly assembled into dimers as observed by Western blotting (data not shown). This confirmed that the observed lack of Na^+^-dependent currents in these mutants is caused by disruption of the substrate binding site and not misfolding of the mutant proteins.

##### Na^+^ and Li^+^ Dependence of the Transient Currents at Different pH Values

Transient currents were recorded for MjNhaP1 proteoliposomes in response to a range of Na^+^ and Li^+^ concentration jumps at pH 7.5, where the currents were high (Figs. 4*B* and Fig. 2, *C* and *D*). Peak currents increased rapidly with substrate concentration, indicating a hyperbolic Michaelis-Menten-type response. Apparent affinity values of the cation binding site were determined by a hyperbolic fit of the Na^+^- and Li^+^-dependent peak currents (Table 1 and Fig. 2, *C* and *D*). The 20-fold increase in affinity for Li^+^ over Na^+^ is in line with other Na^+^/H^+^ exchangers, which have likewise higher affinity for Li^+^ than for Na^+^ (17).

**TABLE 1:** **Kinetic parameters of MjNhaP1** Apparent affinity values (*K_m_*) were determined by a hyperbolic fit of the Na^+^ or Li^+^ dependence of peak currents recorded on an individual SSM sensor and are averages of measurements from three individual sensors (±S.D.). The ratio *k*_2_/*k*_1_ represents the rate constant of H^+^ relative to Na^+^ translocation. Kinetic parameters *k*_2_/*k*_1_, *K_D_*, and p*K* were determined as presented in Fig. 4.

	*K_m_* (pH 7.5)	*K_m_* (pH 6)	*K_D_*	*k*_2_/*k*_1_
	[*mm*]	[*mm*]	[*mm*]	
Na^+^	6.7 ± 0.5	30 ± 1	14	0.2
Li^+^	0.3 ± 0.1	0.9 ± 0.4	0.7	0.5

p*K* (*K*_D_^H^) = 6.8 (1.6·10^−7^ m)				

To investigate whether, as observed in EcNhaA (11), Na^+^ and H^+^ compete for the same binding site in MjNhaP1, experiments were repeated at lower pH. Indeed, concentration jumps at pH 6 revealed a similar competition effect for MjNhaP1 (Table 1 and Fig. 4*B*).

##### Kinetic Model

A valid kinetic model has to account for the experimental substrate dependences of the carrier as well as the observed transient currents. Based on the model shown in Fig. 1*A* the amplitudes of the transient currents (Fig. 4, *A* and *B*), the turnover of the transporter (Fig. 4*C*), and the time dependence of the transient currents (Fig. 4*D*) were numerically calculated, and a consistent parameter set was derived to fit the MjNhaP1 measurements (Fig. 4). From these fits the kinetic parameters for MjNhaP1 were determined as follows: p*K* 6.8, *K*_*D*_^Na^ = 14 mm, *k*_1_ = 350 s^−1^, *k*_2_ = 69 s^−1^ (*k*_2_/*k*_1_ = 0.2) (Table 1). Note that in the framework of the kinetic model these parameters are true binding constants of the binding sites, rather than *K_m_* values.

## DISCUSSION

### 

#### 

##### MjNhaP1 Is a Prototypic Electroneutral Na^+^/H^+^ Exchanger

The NhaP1 Na^+^/H^+^ exchanger from *M. jannaschii* is an important prototype system for the CPA1 family of antiporters, which also includes the pharmacologically relevant mammalian NHE-type Na^+^/H^+^ exchangers. However, activity measurements by fluorescence dequenching with everted vesicles are in themselves insufficient for a detailed analysis of the transport mechanism and kinetics. Limitations arise from the p*K* of the fluorescent dye (10.4), which restricts measurements to a pH above 6. Moreover, the acidification of vesicles with d-lactate by the respiratory chain depends on the outside pH (18). Thus, the pH inside the vesicles and hence the pH gradient established across the membrane in different experiments are not known exactly. In the current study we used SSM-based electrophysiology, which has sufficient time resolution to separate partial steps in the reaction cycle. Therefore, although the overall transport of MjNhaP1 is electroneutral, we were able to isolate the partial electrogenic translocation of Na^+^, Li^+^, or H^+^ within the transport cycle. This is complemented by a Na^+^ efflux technique capable of assessing transporter activity in the pH range 5–8 at well defined inside pH and outside pH.

##### The Kinetic Model Explains the Substrate Dependence of MjNhaP1

In the kinetic model on which our analysis is based (Fig. 1*A*), Na^+^ and H^+^ ions bind alternatively to the carrier in its outward open (C*_o_*) or inward open (C*_i_*) form, whereupon the loaded carrier reorients in the membrane. The model makes two simplifying assumptions: (i) substrate binding equilibria are infinitely fast, and (ii) the carrier is symmetrical, p*K_o_* = p*K_i_*, *K*_*D*,*o*_^Na^ = *K*_*D*,*i*_^Na^, *k*_1_^+^ = *k*_1_^−^, and *k*_2_^+^ = *k*_2_^−^. A carrier is considered symmetrical when transport kinetics in the forward and reverse direction are similar. This has been experimentally confirmed for EcNhaA (11), and we therefore assume the same for MjNhaP1. Additionally, the kinetic model is based on a common binding site for Na^+^ and H^+^. This is confirmed by the competitive behavior of Na^+^ or Li^+^ and H^+^ shown in Table 1, where a decrease in pH results in a decrease of the apparent affinity for Na^+^ or Li^+^ in MjNhaP1. This model yields an excellent fit to all experimental data with only four kinetic parameters. We, therefore, conclude that the model provides an equally appropriate description for MjNhaP1 as previously found for EcNhaA (11).

##### Electrogenic Na^+^ Translocation

**FIGURE 5.**
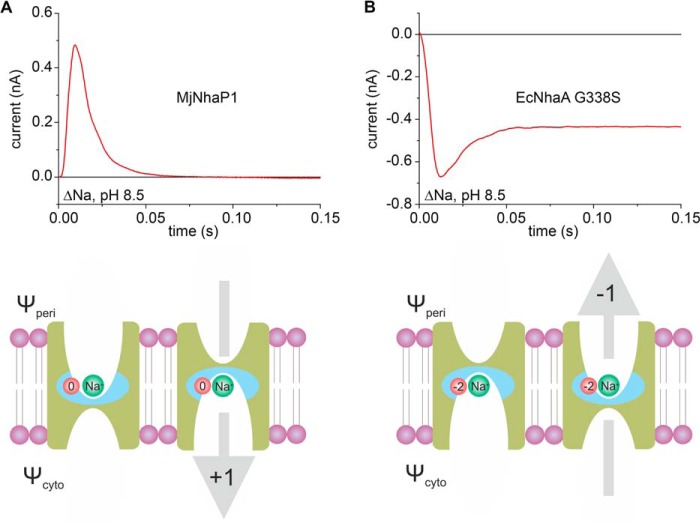
**Na^+^ translocation in MjNhaP1 and EcNhaA.** Transient currents of MjNhaP1 (*A*) and EcNhaA G338S (*B*) were recorded for a Na^+^ concentration jump of 10 mm at pH 8.5. Drawings in *A* and *B* illustrate the mechanism of Na^+^ translocation and the resulting charge displacement in MjNhaP1 (*A*) and EcNhaA (*B*). The indicated direction of substrate translocation portrays the physiological direction of Na^+^ transport in each exchanger. The *blue field* highlights the region of the translocation complex, which comprises charged amino acids (*red circle*) and the transported Na^+^ (*green circle*). Total displaced charge during a Na^+^ translocation event is depicted by the *gray arrow* for each of the two exchangers. Data in the *top panel* of *B* were originally published in Ref. 11.

Na^+^ translocation in MjNhaP1 was found to be associated with a displacement of positive charge. Interestingly, the opposite effect was observed in the CPA2 transporter EcNhaA. Fig. 5 compares both experiments. The high p*K* of EcNhaA (8.8) would require an extremely high pH to separate the Na^+^ translocation step. Therefore, we used the G338S variant, in which the pH profile is shifted to the acidic range (p*K* = 7.0 (11)), close to what we determined for MjNhaP1. For EcNhaA G338S, the current recorded at high pH (Fig. 5*B*) shows a negative transient followed by a stationary current, indicative of electrogenic steady-state transport. MjNhaP1 has a positive current transient (Fig. 5*A*) and lacks the stationary current, as its steady-state transport is electroneutral. Despite the inverse sign of the charge, and accounting for the variability between measurements on different SSM sensors, both current transients have comparable amplitudes, indicating a similar charge displacement.

The *lower panels* in Fig. 5 illustrate how the observed charge displacements in the two transporters may be envisioned. Following substrate binding, a small region of the transporter, the translocation complex (highlighted in *blue* in Fig. 5), is relocated from periplasmic (Ψ_peri_) to cytoplasmic (Ψ_cyto_) potential. The total displaced charge is the sum of charges carried by the substrate ion(s) (one Na^+^ ion is depicted in Fig. 5) and nearby charged amino acid side chains (shown in *red* in Fig. 5). Note that charged side chains that do not change accessibility from one side to the other do not contribute to the charge displacement. Therefore, the region contributing to the charge displacement is likely to be restricted to the substrate binding site and surrounding residues.

In EcNhaA, two negative charges reside in the translocation complex together with the transported Na^+^ ion as shown in Fig. 5*B*. These charges have been proposed to be Asp-163 and Asp-164 (11), which accounts for the observed negative transient current. By contrast, the movement of the substrate-loaded MjNhaP1 from the outside to the inside of the proteoliposomes is associated with a positive charge displacement (Fig. 5*A*). Assuming that the binding site includes the negatively charged Asp-161, both reactions are expected to be electroneutral if one H^+^ or one Na^+^ ion is translocated together with the deprotonated Asp-161 side chain.

Based on these findings we conclude that (i) in addition to the conserved aspartate other charged residues exposed to one side must become accessible to the other side during cation translocation and contribute to the charge displacement. These residues are most likely located at or near the binding site. (ii) Translocation of Na^+^ or H^+^ in MjNhaP1 is accompanied by a net charge displacement of +1, whereas in EcNhaA Na^+^ translocation is associated with a net charge displacement of −1. It remains to be shown whether this is a feature that distinguishes CPA1 from CPA2 transporters. If so, it might reflect a fundamental difference between electroneutral and electrogenic transport. In this context it may be of importance that in the physiological transport direction as shown in the *lower panels* of Fig. 5 the membrane potential acts to speed up Na^+^ translocation in both cases. Thus, further electrophysiological characterizations of other Na^+^/H^+^ exchangers are needed. Most importantly, a high-resolution structure of a substrate-bound Na^+^/H^+^ exchanger is required for a detailed description of the cation binding site.

##### pH Regulation Is an Intrinsic Property of the Na^+^/H^+^ Exchange Mechanism

As discussed in the following section, substrate competition is a simple and effective way to down-regulate CPA2 Na^+^/H^+^ exchangers to prevent overacidification of the cytoplasm. Likewise, CPA1 transporters are down-regulated by substrate depletion to prevent the cytoplasm from becoming too alkaline.

The transport mechanism proposed in Fig. 1*A* is self-regulating, providing an intrinsic means of adaptation to a wide range of physiological conditions. To illustrate this, we simulated different physiological stress conditions, taking into account different ionic conditions and the membrane potential, on the basis of the experimentally determined parameters of the kinetic model and the charge displacements in the Na^+^ and H^+^ translocation steps. For these calculations we used a steady-state model as described previously (11). The results provide insights into the speed of the transport process and are, therefore, superior to simple thermodynamic arguments which can only answer the question whether a process is energetically possible or not.

**FIGURE 6.**
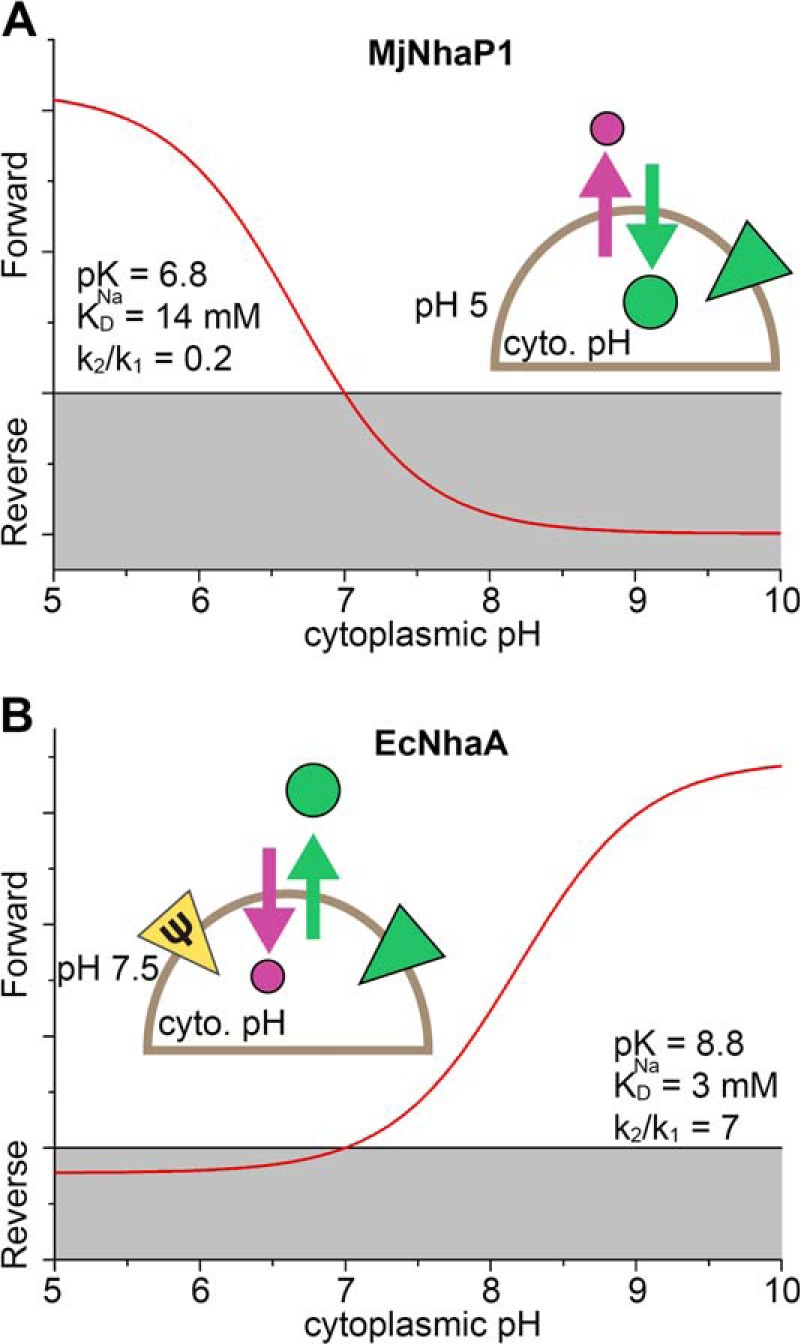
**pH regulation of Na^+^/H^+^ exchangers under physiological conditions.** Calculation was performed using the steady-state solution of the kinetic model shown in Fig. 1 and experimentally determined parameters. *A*, turnover of MjNhaP1 in the *M. jannaschii* cell as a function of cytoplasmic pH under the following conditions: pH_out_ = 5, [Na^+^]_out_ = 500 mm, [Na^+^]_in_ = 5 mm, ΔΨ = −100 mV. Parameters used for the kinetic model were: p*K_o_* = p*K_i_* = p*K* = 6.8, *K*_*D*,*o*_^Na^ = *K*_*D*,*i*_^Na^ = *K*_*D*_^Na^ = 14 mm, *k*_1_^+^ = *k*_1_^−^ = *k*_1_, *k*_2_^+^ = *k*_2_^−^ = *k*_2_, *k*_2_/*k*_1_ = 0.2, displaced charge +1 elementary charge during Na^+^ and H^+^ translocation. *B*, turnover of the EcNhaA Na^+^/H^+^ exchanger in the bacterial *E. coli* cell as a function of cytoplasmic pH under the following conditions: pH_out_ = 7.5, [Na^+^]_out_ = 500 mm, [Na^+^]_in_ = 5 mm, ΔΨ = −150 mV. Parameters used for the kinetic model: p*K_o_* = p*K_i_* = p*K* = 8.8, *K*_*D*,*o*_^Na^ = *K*_*D*,*i*_^Na^ = *K*_*D*_^Na^ = 3 mm, *k*_1_^+^ = *k*_1_^−^ = *k*_1_, *k*_2_^+^ = *k*_2_^−^ = *k*_2_, *k*_2_/*k*_1_ = 7, displaced charge −1 and 0 elementary charges during Na^+^ and H^+^ translocation. *Schematics* show the transport direction of H^+^ (*purple circle* and *arrow*) and Na^+^ (*green circle* and *arrow*) in the “forward” (physiological) mode. The direction of the Na^+^ (*green triangle*) or potential (*yellow triangle*) gradients is also indicated.

The physiological role of NhaP1 in *M. jannaschii* is to control the intracellular pH. To this end the exchanger extrudes H^+^, taking advantage of the sodium gradient, which is most likely maintained by other transporters (19). As a consequence of the low intracellular Na^+^ concentrations on the H^+^ uptake side and the much lower p*K* value compared with EcNhaA, down-regulation due to substrate competition does not occur. Hence, MjNhaP1 remains active at low intracellular pH, allowing the organism to cope with acidic pH stress (Fig. 6*A*). By contrast, as is evident from the lower p*K* value, a rise of the intracellular pH above 7 results in proton depletion. Moreover, the membrane potential accelerates Na^+^ translocation and slows down H^+^ translocation, making this step rate-limiting. Thus, MjNhaP1 is down-regulated at high pH, preventing excessive alkalinization of the cytoplasm.

For comparison, we also calculated the pH-dependent activity of EcNhaA (Fig. 6*B*) using the parameters determined previously (11). NhaA is known to be essential for *E. coli* at elevated Na^+^ concentrations and alkaline pH. Under salt stress conditions, Na^+^ ions are extruded against a Na^+^ gradient. This is possible even at close to neutral external pH because of its electrogenic exchange stoichiometry, which means that Na^+^ transport is accelerated by the membrane potential (11). Conversely, due to substrate competition at the Na^+^ uptake side (inside), the transporter is strongly down-regulated at cytoplasmic pH < 7, preventing acidification of the cell interior.

##### A General Mechanism for Transport and pH Regulation of Na^+^/H^+^ Exchangers

The proposed mechanism provides a simple and elegant explanation of the kinetic properties of the electrogenic CPA2 exchanger EcNhaA (11) and the electroneutral CPA1 exchanger MjNhaP1. It accounts for the widely discussed pH dependence and the observed substrate competition. Moreover, it provides an intrinsic concept of autoregulation (Fig. 1) and can be employed by different organisms to adapt to a wide range of physiological stress conditions. This is easily achieved by changes in affinity constants for Na^+^ or H^+^ or by a change in stoichiometry. Clearly, the model cannot account for some of the reported pH effects in eukaryotic antiporters, such as the up-regulation of Na^+^ efflux (20) or cooperative activation by cytoplasmic acidification (20–23). These are most likely mediated by a large cytoplasmic regulatory domain, which is absent in the prokaryotic Na^+^/H^+^ exchangers. Considering, however, the structural similarity of their transmembrane domain, it seems likely that eukaryotic Na^+^/H^+^ exchangers follow the same mechanistic principles of pH regulation as their prokaryotic ancestors. In particular, alkaline down-regulation by substrate depletion as proposed for MjNhaP1 makes physiological sense. Whether further mechanisms modulate the pH dependence of their activity profile remains to be investigated.

## Footnotes

2 The abbreviations used are:

CPA: cation/proton antiporter family
EcNhaA: NhaA antiporter from *E. coli*
MjNhaP1: NhaP1 antiporter from *M. jannaschii*
NHE: sodium/hydrogen exchanger
SSM: solid-supported membrane.

## References

[1] Brett C. L. Donowitz M. Rao R. (2005) Evolutionary origins of eukaryotic sodium/proton exchangers. Am. J. Physiol. Cell Physiol. 288, C223–239 15643048 10.1152/ajpcell.00360.2004

[2] Padan E. Kozachkov L. Herz K. Rimon A. (2009) NhaA crystal structure: functional-structural insights. J. Exp. Biol. 212, 1593–1603 19448069 10.1242/jeb.026708

[3] Vinothkumar K. R. Smits S. H. Kühlbrandt W. (2005) pH-induced structural change in a sodium/proton antiporter from *Methanococcus jannaschii*. EMBO J. 24, 2720–2729 16015376 10.1038/sj.emboj.7600727 PMC1182236

[4] Goswami P. Paulino C. Hizlan D. Vonck J. Yildiz O. Kühlbrandt W. (2011) Structure of the archaeal Na^+^/H^+^ antiporter NhaP1 and functional role of transmembrane helix 1. EMBO J. 30, 439–449 21151096 10.1038/emboj.2010.321 PMC3025466

[5] Jardetzky O. (1966) Simple allosteric model for membrane pumps. Nature 211, 969–970 5968307 10.1038/211969a0

[6] Shi Y. (2013) Common folds and transport mechanisms of secondary active transporters. Annu. Rev. Biophys. 42, 51–72 23654302 10.1146/annurev-biophys-083012-130429

[7] Forrest L. R. Krämer R. Ziegler C. (2011) The structural basis of secondary active transport mechanisms. Biochim. Biophys. Acta 1807, 167–188 21029721 10.1016/j.bbabio.2010.10.014

[8] Klingenberg M. (1992) in A Study of Enzymes (Kuby S. A., ed) pp. 367–390, CRC Press, Boca Raton, FL

[9] Stein W. D. Honig B. (1977) Models for active transport of cations: steady-state analysis. Mol. Cell. Biochem. 15, 27–44 865483 10.1007/BF01731287

[10] Klingenberg M. (2005) Ligand-protein interaction in biomembrane carriers: the induced transition fit of transport catalysis. Biochemistry 44, 8563–8570 15952762 10.1021/bi050543r

[11] Mager T. Rimon A. Padan E. Fendler K. (2011) Transport mechanism and pH regulation of the Na^+^/H^+^ antiporter NhaA from *Escherichia coli*: an electrophysiological study. J. Biol. Chem. 286, 23570–23581 21566125 10.1074/jbc.M111.230235 PMC3123120

[12] Hellmer J. Teubner A. Zeilinger C. (2003) Conserved arginine and aspartate residues are critical for function of MjNhaP1, a Na^+^/H^+^ antiporter of *M. jannaschii*. FEBS Lett. 542, 32–36 12729893 10.1016/s0014-5793(03)00332-6

[13] Schulz P. Garcia-Celma J. J. Fendler K. (2008) SSM-based electrophysiology. Methods 46, 97–103 18675360 10.1016/j.ymeth.2008.07.002

[14] Bazzone A. Costa W. S. Braner M. Cǎlinescu O. Hatahet L. Fendler K. (2013) Introduction to solid supported membrane-based electrophysiology. J. Vis. Exp. 75, e50230 23711952 10.3791/50230 PMC3679796

[15] Hellmer J. Pätzold R. Zeilinger C. (2002) Identification of a pH-regulated Na^+^/H^+^ antiporter of *Methanococcus jannaschii*. FEBS Lett. 527, 245–249 12220668 10.1016/s0014-5793(02)03244-1

[16] Garcia-Celma J. J. Hatahet L. Kunz W. Fendler K. (2007) Specific anion and cation binding to lipid membranes investigated on a solid supported membrane. Langmuir 23, 10074–10080 17718523 10.1021/la701188f

[17] Lee C. Kang H. J. von Ballmoos C. Newstead S. Uzdavinys P. Dotson D. L. Iwata S. Beckstein O. Cameron A. D. Drew D. (2013) A two-domain elevator mechanism for sodium/proton antiport. Nature 501, 573–577 23995679 10.1038/nature12484 PMC3914025

[18] Reenstra W. W. Patel L. Rottenberg H. Kaback H. R. (1980) Electrochemical proton gradient in inverted membrane vesicles from *Escherichia coli*. Biochemistry 19, 1–9 6986161 10.1021/bi00542a001

[19] Thauer R. K. Kaster A. K. Seedorf H. Buckel W. Hedderich R. (2008) Methanogenic archaea: ecologically relevant differences in energy conservation. Nat. Rev. Microbiol. 6, 579–591 18587410 10.1038/nrmicro1931

[20] Wakabayashi S. Hisamitsu T. Pang T. Shigekawa M. (2003) Kinetic dissection of two distinct proton binding sites in Na^+^/H^+^ exchangers by measurement of reverse mode reaction. J. Biol. Chem. 278, 43580–43585 12928437 10.1074/jbc.M306690200

[21] Fuster D. Moe O. W. Hilgemann D. W. (2008) Steady-state function of the ubiquitous mammalian Na/H exchanger (NHE1) in relation to dimer coupling models with 2Na/2H stoichiometry. J. Gen. Physiol. 132, 465–480 18824592 10.1085/jgp.200810016 PMC2553392

[22] Hisamitsu T. Yamada K. Nakamura T. Y. Wakabayashi S. (2007) Functional importance of charged residues within the putative intracellular loops in pH regulation by Na^+^/H^+^ exchanger NHE1. FEBS J. 274, 4326–4335 17662110 10.1111/j.1742-4658.2007.05962.x

[23] Lacroix J. Poët M. Maehrel C. Counillon L. (2004) A mechanism for the activation of the Na/H exchanger NHE-1 by cytoplasmic acidification and mitogens. EMBO Rep. 5, 91–96 14710192 10.1038/sj.embor.7400035 PMC1298952

